# Supplementary material for: Stroke and TIA Survivors’ Perceptions of the COVID-19 Vaccine and Influences on Its Uptake: Cross Sectional Survey
Source: Int J Environ Res Public Health. 2022 Oct 25;19(21):13861. doi: 10.3390/ijerph192113861 (PMC9658254; doi:10.3390/ijerph192113861)
Supplement: Supplementary file 1 [file ijerph-19-13861-s001.zip › Supplementary Materials File S1.pdf]

Perspectives of the COVID-19 vaccine: people who have had a stroke or mini stroke

Information about you

Have you had a stroke or mini stroke (transient ischemic attack: TIA)?

- ☐ Stroke
- ☐ Mini-stroke (transient ischemic attack: TIA)
- ☐ Both
- ☐ Unsure

Are you:

- ☐ Male
- ☐ Female
- ☐ Non-binary
- ☐ Prefer not to say

What is your age?

- ☐ 18 - 25 years
- ☐ 26 - 35 years
- ☐ 36 - 45 years
- ☐ 46 - 55 years
- ☐ 56 - 65 years
- ☐ 66 - 75 years
- ☐ 76 - 85 years
- ☐ 86 - 95 years
- ☐ ≥100 years

What is your ethnicity?

- ☐ White
- ☐ Mixed/ multiple ethic groups
- ☐ Asian/ Asian British
- ☐ Black/ African/ Caribbean/ Black British
- ☐ Prefer not to say
- ☐ Other (please specify):

What is your highest level of education?

- ☐ No formal qualification
- ☐ GCSE / O-Levels / CSE / Foundation Diploma
- ☐ Apprenticeship
- ☐ AS / A-Levels / BTEC / Advanced NVQs
- ☐ Degree (e.g. BA/BSc)
- ☐ Higher Degree (e.g. MSc/PhD)
- ☐ Other (please specify):

What is your current employment status?

- ☐ Employed- full time
- ☐ Employed- part time
- ☐ Furloughed- full time
- ☐ Furloughed- part time
- ☐ Volunteer
- ☐ Housewife/ househusband
- ☐ Unemployed
- ☐ Retired
- ☐ Other (please specify):

Have you had COVID-19?

- ☐ Yes
- ☐ No
- ☐ Unsure

COVID-19 vaccination

Have you had the COVID-19 vaccine?

- [ ] Yes- first dose
- [ ] Yes- first and second dose
- [ ] No- I’ve not been offered it yet
- [ ] No- I declined the vaccine
- [ ] Other (please specify):

Any comments:

Why did you decline?

Why did you decline the vaccine? Tick all that apply

- [ ] Concerned about side effects of the vaccine
- [ ] Concerned about interaction of the vaccine with stroke prevention medication
- [ ] Concerned the vaccine is too new
- [ ] Fear of needles
- [ ] Don’t believe in vaccines in general
- [ ] Prefer not to say
- [ ] Other (please specify):

Comments:

How likely are you to accept the vaccine?

If you haven't been offered it yet, how likely are you to accept the vaccine when it is offered to you?

- ☐ Definitely will accept it
- ☐ Very likely to accept it
- ☐ Likely to accept it
- ☐ Unlikely to accept it
- ☐ Very unlikely to accept it
- ☐ Definitely not accept it

Any comments?

Perspectives of COVID-19 vaccine: safety

To what extent do you agree or disagree with the following statements. I am concerned about:

	Strongly agree	Agree	Neither agree nor disagree	Disagree	Strongly disagree	Not applicable
The safety of the vaccine.	☐	☐	☐	☐	☐	☐
Side effects of the vaccine.	☐	☐	☐	☐	☐	☐
How new the vaccine is.	☐	☐	☐	☐	☐	☐
The vaccine causing an anaphylactic shock (severe allergic reaction).	☐	☐	☐	☐	☐	☐
The vaccine affecting my blood thinning medication (e.g. anticoagulants, warfarin).	☐	☐	☐	☐	☐	☐
The vaccine affecting my cholesterol lowering medication (e.g. statins)	☐	☐	☐	☐	☐	☐
The vaccine affecting my blood pressure lowering medication (e.g. antihypertensives)	☐	☐	☐	☐	☐	☐
The vaccine increasing my stroke risk.	☐	☐	☐	☐	☐	☐

	Strongly agree	Agree	Neither agree nor disagree	Disagree	Strongly disagree	Not applicable
I have a fear of needles.	☐	☐	☐	☐	☐	☐
I have other concerns about the vaccine (please specify in comments box).	☐	☐	☐	☐	☐	☐

Any comments?

Perspectives of COVID-19 vaccine: access to the vaccine appointment

To what extent do you agree or disagree with the following statements:

	Strongly agree	Agree	Neither agree nor disagree	Disagree	Strongly disagree	Not applicable
I understand how to get the vaccine, such as where to go and how to get there.	☐	☐	☐	☐	☐	☐
I have difficulty accessing the vaccination appointment (e.g. unable to travel).	☐	☐	☐	☐	☐	☐
I do not have the time to attend the vaccination appointment.	☐	☐	☐	☐	☐	☐
I need to see my doctor before I attend the vaccination appointment.	☐	☐	☐	☐	☐	☐

Any comments?

Beliefs and social influences

To what extent do you agree or disagree with the following statements:

	Strongly agree	Agree	Neither agree nor disagree	Disagree	Strongly disagree	Not applicable
I believe the vaccine will protect me against COVID-19.	☐	☐	☐	☐	☐	☐
I believe having the vaccine will help reduce the spread of Covid-19.	☐	☐	☐	☐	☐	☐
I know other people who have had the vaccine.	☐	☐	☐	☐	☐	☐
I believe having the vaccine is the right thing to do.	☐	☐	☐	☐	☐	☐
Religious or cultural beliefs impact my decision to have the vaccine.	☐	☐	☐	☐	☐	☐
In general, I disagree with or don’t trust vaccines	☐	☐	☐	☐	☐	☐

Any comments?

Knowledge and understanding

To what extent do you agree or disagree with the following statements:

	Strongly agree	Agree	Neither agree nor disagree	Disagree	Strongly disagree	Not applicable
I am satisfied with my knowledge and understanding of the vaccine.	☐	☐	☐	☐	☐	☐
I understand where stroke and mini stroke (TIA) patients are on the vaccination priority list.	☐	☐	☐	☐	☐	☐
I have searched for information about the vaccine for people who have had a stroke/ mini stroke (TIA).	☐	☐	☐	☐	☐	☐
I am satisfied with the information I found about the vaccine for people who have had a stroke/ mini stroke (TIA).	☐	☐	☐	☐	☐	☐

Any comments?

Have you used any of the resources or people below to find information about the COVID-19 vaccine for people who have had a stroke/ mini stroke (TIA)? Tick all that apply.

- ☐ Google
- ☐ Government website
- ☐ NHS website
- ☐ Stroke Association website
- ☐ Social media
- ☐ GP
- ☐ Stroke doctor
- ☐ Stroke Association worker
- ☐ Other (please specify):

Any comments?

COVID-19

To what extent do you agree or disagree with the following statements:

	Strongly agree	Agree	Neither agree nor disagree	Disagree	Strongly disagree	Not applicable
I will get very sick if I get COVID-19.	☐	☐	☐	☐	☐	☐
I am at greater risk of COVID-19 related complications due to my stroke/TIA	☐	☐	☐	☐	☐	☐
I am at high risk of getting COVID-19.	☐	☐	☐	☐	☐	☐
If I get COVID-19, I am likely to pass it on to other people.	☐	☐	☐	☐	☐	☐
I believe that having COVID-19 will increase my chances of further stroke/TIA.	☐	☐	☐	☐	☐	☐

Any comments?
