# Supplementary material for: Stroke and TIA Survivors’ Perceptions of the COVID-19 Vaccine and Influences on Its Uptake: Cross Sectional Survey
Source: Int J Environ Res Public Health. 2022 Oct 25;19(21):13861. doi: 10.3390/ijerph192113861 (PMC9658254; doi:10.3390/ijerph192113861)
Supplement: Supplementary file 1 [file ijerph-19-13861-s001.zip › Supplementary Materials File S2.pdf]

Survey Advert

Example survey advert

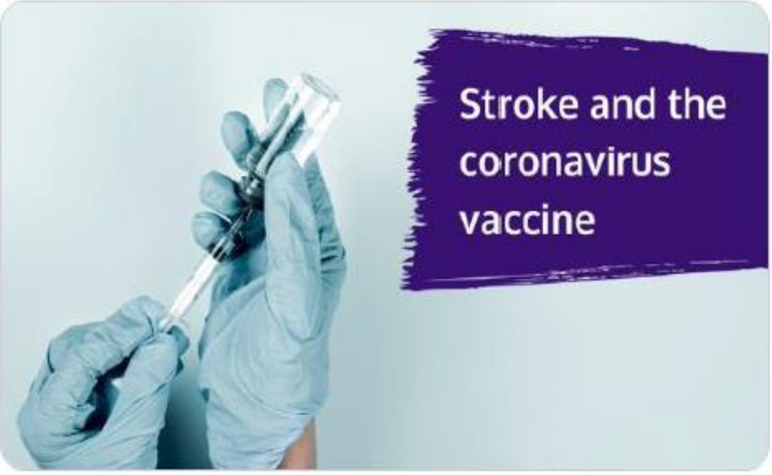

We're inviting people who have had a stroke or mini-stroke (TIA) to complete a **survey about the COVID-19 vaccine**.

We want to understand how you feel about the COVID-19 vaccine and if you have any concerns.

Please complete this survey to share your views: https://strk.org.uk/3pSNxOJ

Stroke Association | UNIVERSITY OF BIRMINGHAM | INSTITUTE OF APPLIED HEALTH RESEARCH
